# Sensitivity Analysis of One-Dimensional Multiphysics Simulation of CO_2_ Electrolysis Cell

**DOI:** 10.1021/acs.jpcc.4c00690

**Published:** 2024-06-27

**Authors:** Harry Dunne, Weiming Liu, Mohammad Reza Ghaani, Kim McKelvey, Stephen Dooley

**Affiliations:** † School of Physics, Trinity College Dublin, Dublin D02 PN40, Ireland; ‡ MacDiarmid Institute for Advanced Materials and Nanotechnology and School of Chemical and Physical Sciences, Victoria University of Wellington, Wellington 6012, New Zealand

## Abstract

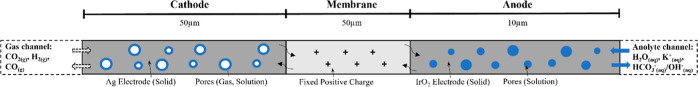

Electrochemical (EC) carbon dioxide (CO_2_) reduction, where CO_2_ is converted to value-added products such as fuel precursors, plays a key role in helping the world’s energy system reach net-zero carbon emissions. Simulations of EC cells provide valuable insight into their operation since detailed experimental results on short length and time scales are difficult to obtain. In this work, we construct a 1D simulation of a membrane-electrode-assembly EC cell for CO_2_ reduction, using a porous silver gas diffusion cathode. We run the simulation under different electrolyte conditions, showing how the cell performance is affected. We then perform a sensitivity analysis of all input parameters to the simulation, which has not been presented before in the literature. We show that the CO partial current density (*i*_CO_) is significantly affected by each input parameter of the simulation. *i*_CO_ is most sensitive to EC kinetic parameters (*i*_0_/α) of all EC reactions, with a 1% change in α resulting in up to 6% change in *i*_CO_. Since there is uncertainty associated with the value of each input parameter, this indicates that infidelity between experiment and simulation is likely, and thus, caution should be practiced when comparing experimental results to simulation results. Further, we show that the large range of conditions simulated in literature helps to explain the large variance in reported values of *i*_0_ and α. The results of this paper demonstrate the potential of sensitivity analysis methods to quickly optimize aspects of cell performance (CO_2_ utilization, Faradaic efficiency, etc.).

## Introduction

### Context

The field of electrochemical (EC) CO_2_ reduction is concerned with converting CO_2_ to value-added products by means of EC reactions. The field is undergoing a surge in popularity as increasing focus is shifting to net-zero-emission energy systems for the world in light of the climate crisis. EC CO_2_ reduction creates a source of renewable hydrocarbons, eliminating the need for fossil resources, if the value-added products are fuels or fuel precursors. It incentivizes carbon capture by turning captured CO_2_ to value-added products, and it is also a way of storing excess renewable energy in the form of hydrocarbons for later use.^1^

The operation of a CO_2_ electrolysis cell is complex, as gas flow, liquid flow, EC reactions, solution-phase chemical reactions, phase change of species between gas and liquid phases, electron transport, and adsorption of species onto the catalyst surface all occur simultaneously and influence each other. For this reason, it is difficult to operate these cells consistently and reliably at high current densities with high efficiencies.^2,3^ To aid with this problem, simulations of CO_2_ electrolyzers have been increasing in number, as can be seen in the review of Bui et al.^4^ Such simulations, or “digital twins” to experiment, are useful as they provide insight at length and time scales which are impossible or difficult to attain by experiment. The purpose of such simulations is to characterize the output of the cell, typically the current–voltage behavior, and the quantities of chemical species in the cell as a function of time and operating conditions.

In this work, we describe a multiphysics simulation of a zero-gap membrane-electrode-assembly (MEA) cell, with an anion exchange membrane (AEM) and a porous silver (Ag) gas-diffusion electrode (GDE) as the cathode, of which several 1D simulations exist already in the literature.^5−13^ MEA cells with a GDE cathode have been shown to produce higher current densities primarily due to higher rates of mass transport to the cathode surface caused by a shorter diffusion path for CO_2_ to the electrode surface.^11^

### Literature Review

Due to constraints of time and computational power, multiphysics simulations (which are mostly in 1D) simulate some, but not all, of the physics occurring inside a real electrolysis cell. Simulations generally consider (i) transport of liquid-phase species, (ii) transport of gas-phase species, (iii) transport of electrons in solid phase, (iv) EC reaction kinetics at the electrode surface, and (v) solution-phase reaction kinetics in the electrolyte.^4^ The equations used are implemented often in the same simulation platform, COMSOL Multiphysics, although other platforms are available, e.g., ANSYS-Fluent. From these numerical frameworks, cell behavior under certain conditions and using different setups have been assessed and hypothesized. For instance, Singh et al.^6^ investigate different sources of voltage loss in a photovoltaic (PV) cell and propose optimal device configurations to minimize them—suggesting the use of an AEM to minimize overall potential losses, as this allows transport of anions which are the primary charge carriers in this system. This finding is backed up by Weng et al.^11^ who simulate three different cases: a full MEA (gas feed at anode and cathode) and an exchange MEA (liquid feed at anode, gas feed at cathode) using either KOH or KHCO_3_ as the electrolyte. Wang et al.^14^ also simulate this setup and show that the CO_2_ conversion efficiency is limited to ∼50%, due to crossover of carbonate anions across the membrane from cathode to anode and subsequent conversion to CO_2_, which leaves the cell unreacted.

CO_2_ reduction simulations are growing in sophistication, following the trend of water reduction simulations.^4^ Earlier works simulated half-cells,^9,12,15,16^ often only considering electrodes as planar electrodes (i.e., treating electrodes as a 0D surface), but now more consider full cells (anode and cathode), with electrodes treated as porous structures.^6−8,10,11,13^ Some 2D simulations of half-cells have been developed,^17−23^ which give deeper insight into cell behavior as they allow us to examine perpendicular flow, as well as distribution of current and species in two dimensions. Many studies do not consider a full set of solution-phase reactions, as this presents the challenge of knowing the reaction kinetic constants as well as making model convergence more difficult.^4^ Simplifying assumptions have been made, for instance, Moore et al.^18^ impose a concentrated KOH solution with pH above 12 and thus neglect the carbonate/bicarbonate buffer reactions (reactions R4, R5 and R7 in this work) and consider consumption of CO_2_ by OH^–^ (reaction R6) to be irreversible. The inclusion of a full solution-phase reaction system is advised, as carbonate/bicarbonate anions are identified by Rabinowitz and Kanan,^24^ as well as Wang et al.,^14^ as limiting cell performance by reducing the carbon efficiency as will be discussed in the next section.

One key challenge in building a multiphysics simulation of a CO_2_ electrolysis cell is sourcing the input parameters. Many input parameters needed for multiphysics simulations are difficult and time-consuming to measure (e.g., active surface areas of porous electrodes). For this reason, those building simulations often source parameters from work done by other groups. However, the conditions under which these parameters were originally determined do not always exactly match those of the system being simulated. This will lead to “infidelity” in the simulation—the output of the simulation will be different to that of the experiment to which it is being compared. Further, many of the input parameters commonly used may be inaccurate. For example, those who do include solution-phase reactions generally use the values presented by Bui et al.,^4^ or Schulz et al.^25^ for their rate constants and do not account for the fact that rate constants change with conditions, e.g., under electric fields. It is common practice to round these parameters to the nearest order of magnitude from their reference source. The problem of sourcing parameters becomes more difficult since many parameters are highly variable. One example is the EC kinetic parameters, *i*_0_ and α, used in the Butler–Volmer equations, which are used to calculate current density of an EC reaction. For every reaction, there is a large variance in the reported values for *i*_0_ and α. Table 1 shows the reported *i*_0_ and α values for reaction R1, CO_2_ reduction to CO. These parameters are usually extracted by fitting the Butler–Volmer equations to experimental data. This is an overly simplistic method however as it does not consider effects such as mass transport, which have affected the experimental data, as pointed out by Corpus et al.^26^ They suggest fitting the output of a multiphysics simulation to experimental data, thus accounting for mass transport effects and effects of solution-phase reactions.

**Table 1:** Range in Reported EC Kinetic Parameters *i*_0,1_ and α_c,1_ from Published Multiphysics Simulations of CO_2_ EC Reduction Cells Using Ag Cathodes^a^

authors/year	*i*_0,1_: exchange current density for reaction R1 (mA/cm^2^)	α_c,1_: cathodic transfer coefficient for reaction R1 (−)	electrolyte [conc.]	cell type		membrane	products considered (pathway)
Gutierrez and Haussener 2016^21^	4.68 × 10^–1^	0.14	KOH [varied]	PV cell	full cell	neutral or anion exchange	CO, H_2_ (basic), O_2_ (acidic)
Gutierrez and Haussener 2016^21^	3.89 × 10^–1^	0.13	NaOH [varied]	PV cell	full cell	neutral or anion exchange	CO, H_2_(basic), O_2_ (acid)
Yang et al. 2020^19^	7.08 × 10^–2^	0.40	KOH [0.1–2 M]	GDE	half cell		CO, H_2_ (basic), HCOO^–^
Delacourt and Newman 2010^30^	1.12 × 10^–3^	0.50	KHCO_3_ [0.5 M]	MEA, buffer layer, GDE	full cell	cation exchange	CO, H_2_ (basic), O_2_ (basic)
Weng et al. 2018^9^	4.68 × 10^–4^	0.44	KHCO_3_ [0.5 M]	GDE	half cell		O_2_, H_2_ (acidic), H_2_ (basic)
Singh et al. 2015^6^	1.51 × 10^–4^	0.29	varied [varied]	PV cell	full cell	cation/anion exchange	O_2_, CO
Singh et al. 2016^7^	4.38 × 10^–5^	0.341	varied [varied]	planar	full cell	anion exchange	CO, H_2_ (basic), O_2_ (acidic)
Kas et al. 2021^17^	3.31 × 10^–5^	0.33	KHCO_3_ [1 M]	GDE	half cell		CO, H_2_ (basic)
Singh et al. 2017^8^	1.71 × 10^–5^	0.49	KHCO_3_ [0.1 M]	planar	full cell	anion exchange	CO, H_2_ (basic), O_2_ (acidic)
Suter and Haussener 2019^28^	1.00 × 10^–6^	0.29	KHCO_3_ [0.1 M]	planar	half cell		CO, H_2_ (basic)
Suter and Haussener 2019^28^	8.32 × 10^–7^	0.29	KHCO_3_ [0.1 M]	planar	half cell		CO, H_2_ (basic)
Agliuzza et al. 2023^22^	7.20 × 10^–8^	0.25277/0.23708	KHCO_3_ [0.1 M]	planar	full cell	cation exchange	CO, H_2_ (acidic), O_2_ (acidic)
Weng et al. 2019^11^	2.14 × 10^–9^	1.00	KHCO_3_ [0.5 M], KOH [0.5 M]	multiple cells simulated	full cell	anion exchange	CO, H_2_ (acidic), H_2_ (basic), O_2_ (acidic), O_2_ (basic)
Petrov et al. 2023^23^	3.48 × 10^–14^	1.00	KHCO_3_ [varied]	GDE	full cell	anion exchange	CO, H_2_ (basic), O_2_ (acidic), O_2_ (basic)

a There is a large range in the reported *i*_0_/α values, corresponding to the large range in simulated conditions.

Published multiphysics simulations are summarized in Table 1. Approximately one-third are not validated experimentally,^6,10,11,17,18,21,27^ as it is difficult to obtain experimental data aside from polarization curves or Faradaic efficiencies. One-third of simulations validate their simulation using data from other published experimental setups.^8,9,12,13,15,28,29^ These setups, however, may not be representative of the simulated system. One-third of simulations are validated by experiments performed by the same group.^7,16,19,20,22,23,30−32^

Table 1 shows the range in reported EC kinetic parameters *i*_0,1_ and α_c,1_. Note that each case simulates a different cell setup under different operating conditions, thus corresponding to different input parameters. The choice of cell configuration, electrolyte, membrane, full or half-cell, and reactions considered are all different and thus help to explain the large discrepancy in the reported values of *i*_0,1_ and α_c,1_. Each different condition results in a different set of input parameters used by the simulation, which affects the output of the simulation and, thus, the fitting process.

In the Supporting Information, we include a further discussion of each input parameter, where they are usually sourced, and how they may lead to infidelity in the output of a simulation and thus to extraction of inaccurate kinetic parameters.

### Objectives of This Work

In this work, we aim to investigate the effects of each input parameter on the current density of the CO_2_ reduction reaction, *i*_CO_. To this end, we construct a multiphysics simulation of a full EC cell for CO_2_ reduction, considering chemical reactions and mass transport. We consider EC reactions, reversible solution-phase reactions, and solvation of CO_2__(g)_ to CO_2__(aq)_. Mass transport considered is of chemical species in the gas and solution phases and of electrons in the solid phase. We then perform sensitivity analysis of the simulation input parameters to assess how they affect the output of the simulation. This sensitivity analysis helps to explain the large variation in reported values of *i*_0_ and α. It also suggests where more caution should be observed in sourcing input parameters to simulations of this type to minimize infidelity between experiment and simulation. Sensitivity analysis also informs future experiments by telling us what we can change in the cell to improve performance most easily.

## Methodology

### Overview of Simulation

**Figure 1:**
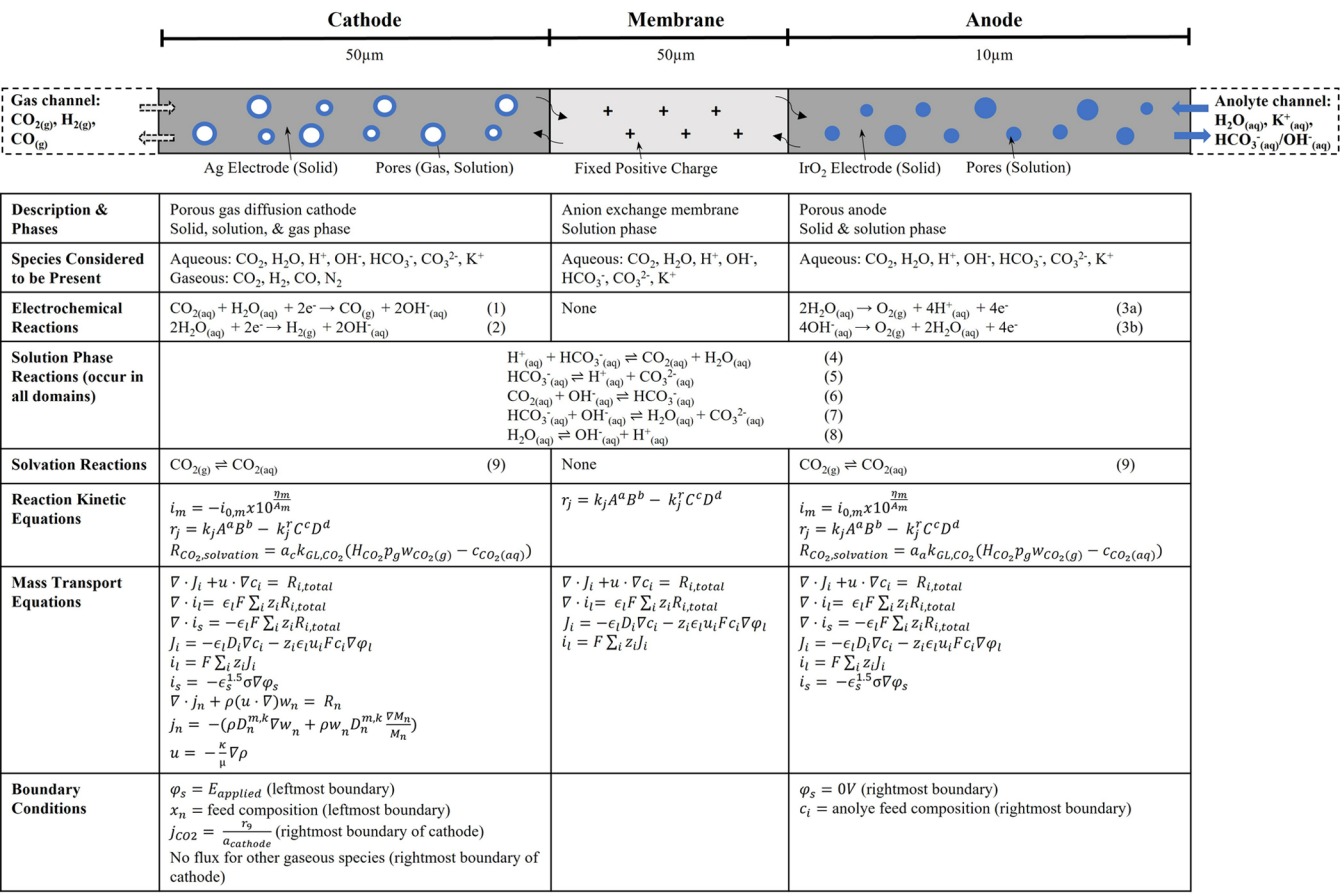
Three domains (cathode, membrane, and anode) in the 1D simulation of the CO_2_ electrolysis cell presented in this work. Below the 1D domains simulated, the MEA cell setup is illustrated. The cell consists of a cathode (a GDE of pure porous silver, i.e., pure catalyst), an anion-exchange membrane, and an anode (carbon paper coated with an IrO_2_ catalyst layer). At the leftmost boundary (gas channel/cathode boundary), we fix the mole fraction of CO_2(g)_ corresponding to inlet CO_2(g)_ feed. At the rightmost boundary (anode/electrolyte channel boundary), we fix the concentrations of solution-phase species H_2_O_(aq)_, H_(aq)_^+^, OH_(aq)_^–^, HCO_3__(aq)_^–^, CO_3(aq)_^2–^, and K_(aq)_^+^, corresponding to delivery of anolyte solution (either 0.1 M KHCO_3_ or 0.1 M KOH). The table below the illustration shows the species and reactions in each domain, the main equations used to simulate reaction kinetics and transport, and the boundary conditions used. All equations and terms are described in the Methodology section. The equations are populated with parameters from Table 2, and they are solved using COMSOL Multiphysics 6.0. Equations are described in Newman.^33^

The one-dimensional multiphysics simulation presented in this work is composed of three domains as shown in Figure 1: cathode, anion-exchange membrane, and anode. The simulation accounts for EC (surface) reactions, solution-phase reactions, solvation of CO_2(g)_ to CO_2(aq)_, transport of gaseous species (CO_2(g)_, H_2(g)_, CO_(g)_, N_2(g)_) and solution-phase species (CO_2(aq)_, H_2_O_(aq)_, H_(aq)_^+^, OH_(aq)_^–^, HCO_3(aq)_^–^, CO_3__(aq)_^2–^, and K_(aq)_^+^), and transport of electrons in the solid phase. Both electrodes are described as porous electrodes, meaning reactions and transport occur simultaneously in these domains. In the cathode domain, transport of gas- and solution-phase species occurs alongside reaction kinetics, as the cathode is a GDE. In the anode, transport of solution-phase species occurs alongside reaction kinetics. The equations in Figure 1 applied to each domain, governing transport and kinetics, are outlined below and are also outlined in the review of Bui et al.^4^ The equations are populated with parameters in Table 2 and solved using COMSOL Multiphysics v6.0.

**Table 2:** Input Parameters Provided to the 1D Simulation^a^

parameter	value	unit	references
*i*_0,1_	1 × 10^–3^	mA cm^–2^	this work
*i*_0,2_	1 × 10^–7^	mA cm^–2^	this work
*i*_0,3_	1 × 10^–8^	mA cm^–2^	this work
α_c,1_	0.4		this work
α_c,2_	0.6		this work
α_c,3_	0.5		this work
*E*_0,1_	–0.11	V	(11)
*E*_0,2_	0	V	(11)
*E*_0,3_	1.23	V	(11)
*k*_4_	3 × 10^–2^	M s^–1^	(4)
*k*_5_	59	M s^–1^	(4)
*k*_6_	2 × 10^3^	M s^–1^	(4)
*k*_7_	6 × 10^9^	M s^–1^	(4)
*k*_8_	1 × 10^–3^	M s^–1^	(4)
*K*_eq 4_	4 × 10^–7^		(4)
*K*_eq 5_	4 × 10^–11^		(4)
*K*_eq 6_	4 × 10^7^		(4)
*K*_eq 7_	4 × 10^3^		(4)
*K*_eq 8_	1 × 10^–14^		(4)
*D*_CO_2__	1.910 × 10^–5^	cm^2^ s^–1^	(9)
*D*_CO_3_^2–^_	0.923 × 10^–5^	cm^2^ s^–1^	(9)
*D*_H^+^_	9.310 × 10^–5^	cm^2^ s^–1^	(9)
*D*_H_2_O_	2.299 × 10^–5^	cm^2^ s^–1^	(34)
*D*_HCO_3_^–^_	1.185 × 10^–5^	cm^2^ s^–1^	(9)
*D*_K^+^_	1.957 × 10^–5^	cm^2^ s^–1^	(9)
*D*_OH^–^_	5.293 × 10^–5^	cm^2^ s^–1^	(9)
*D*_CO_2_–N_2__	0.165	cm^2^ s^–1^	(9)
*D*_CO–N_2__	0.202	cm^2^ s^–1^	(9)
*D*_CO–CO_2__	0.152	cm^2^ s^–1^	(9)
*D*_H_2_–CO_2__	0.646	cm^2^ s^–1^	(9)
*D*_H_2_–N_2__	0.779	cm^2^ s^–1^	(9)
*D*_H_2_–CO_	0.743	cm^2^ s^–1^	(9)
*L*_cathode_	50	μm	this work
*L*_membrane_	50	μm	this work
*L*_anode_	10	μm	this work
σ_anode_	100	S m^–1^	(9)
σ_cathode_	100	S m^–1^	(9)
ρ_mem_	2 × 10^8^	C m^–3^	(9)
ϵ_l,anode_	0.6		this work
ϵ_l,cathode_	0.6		this work
ϵ_l,membrane_	0.6		this work
*a*_anode_	1 × 10^6^	m^–1^	(9)
*a*_cathode_	1 × 10^6^	m^–1^	(9)
δ_TF_	10	nm	(9)
*r*_p_	0.25	μm	(9)
*p*_gas_	1	atm	this work
temperature	298	K	this work
*x*_CO_2__	0.99		this work
*H*_CO_2__	3.1 × 10^–2^	M atm^–1^	(35)
*E*_applied_	1–3.5	V	this work

a See the Results section for a discussion on the effect of each parameter on the output of the simulation based on the results of the sensitivity analysis.

### Electrochemical, Solution-Phase, and Solvation Reactions Simulated

The EC reactions occurring at the cathode are the formation of CO_(g)_ and the unwanted formation of H_2(g)_
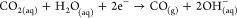
R1
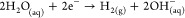
R2

At the anode, the formation of O_2_(g)__ occurs due to water oxidation, which occurs via two pathways
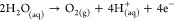
R3a
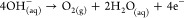
R3b

These EC reactions are commonly used to describe CO_(g)_, H_2(g)_, and O_2(g)_ formation on Ag and IrO_2_ catalysts,^4^ and are commonly referred to as CO_2_R, HER, and OER reactions, respectively (i.e., CO_2_ reduction reaction, hydrogen evolution reaction, oxygen evolution reactions). As is the standard in multiphysics simulations, these reactions are approximated as a single step. This approximation is a source of infidelity between experiment and simulation^8^ (see discussion in Results). Note that this study excludes the acidic pathway of the HER reaction [2H^+^ + 2e^–^ → H_2_] due to the alkaline conditions simulated and the resulting low H^+^ concentration. The solution-phase reactions simulated are the bicarbonate/carbonate system reactions (reactions R4–R7) and the dissociation of H_2_O_(aq)_ (reaction R8), listed below:
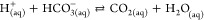
R4
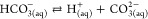
R5
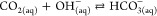
R6
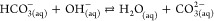
R7
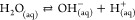
R8

The solvation of CO_2(g)_ to CO_2(aq)_ within the cathode is also simulated:
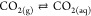
R9

CO_2_ is the only species existing in both gas and solution phases in the simulation—gas-phase water and effects, e.g., those due to humidity, are excluded since the temperature of the system is 298 K. The solution-phase reactions outlined above are those which are commonly included in multiphysics simulations of CO_2_ reduction cells and whose apparent rate constants have been studied,^25^ although we note that there may be reactions occurring and influencing cell performance, which are not listed here. In the Supporting Information, we include a list of such reactions.

It is suggested that the CO_2_ utilization efficiency of a zero-gap MEA cell with anion-exchange membranes is limited to ∼50% due to the consumption of CO_2(aq)_ at the cathode by reaction R6, forming carbonates (HCO_3_^–^/CO_3_^2–^). It is also suggested that CO_2_ is formed at the anode due to crossover of these carbonate anions through the membrane to the anode side and their subsequent conversion back to CO_2(aq)_ at the anode.^14,24^

### Kinetics Governing Chemical Reactions

Reactions R1–R3b are governed by the Tafel equation:
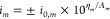
1where *i*_*m*_ is the current density of EC reaction *m*, *i*_0,*m*_ is the exchange current density. The Tafel slope *A*_*m*_ is given by
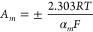
2where *R* is the universal gas constant, *T* is the temperature, and *a*_*m*_ is the anodic or cathodic kinetic fitting parameter for reaction *m*. The overpotential η_*m*_ is given by
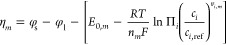
3where φ_s_ and φ_l_ are the potentials in the solid and liquid phases, respectively, *E*_0,*m*_ is the standard reduction potential, *v*_*i*_,_*m*_ is the stoichiometric coefficient of species *i* in reaction *m*, *c*_*i*_ is the concentration of species *i*, *n*_*m*_ is the number of electrons transferred, and *F* is Faraday’s constant. For eqs 1 and 2, the signs are positive for an anodic reaction and negative for a cathodic reaction. All EC reactions are summed over to give the total rate of production/consumption for each species *i* according to
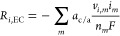
4where *a*_(c/a)_ is the specific surface area of the cathode/anode.

For solution-phase reactions (reactions R4–R8), forward rate constants and equilibrium constants *k*_*j*_, *K*_eq_,_*j*_ of each reaction *j* are supplied as input parameters, and reverse rate constants calculated according to
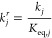
5

The rate *r*_*j*_ of each solution-phase reaction *j* is defined as
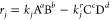
6where A–D represent activities of the species involved and *a*–*d* their stoichiometric coefficients (the activity of a species is its concentration divided by 1 M or 55 M in the case of water^4^). For each species, the rate of production/consumption is calculated by summing over all of the reactions it is involved in:
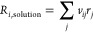
7

CO_2(aq)_ is the reactant in reaction R1 rather than CO_2(g)_ since it is suggested that H_(aq)_^+^ ions are needed for the reaction to occur.^7^ We follow the method of Weng et al.^9^ and others and define within the cathode the rate of reaction R9 as
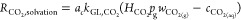
8where  is the gas to liquid mass transfer coefficient, (δ_TF_ is the thickness of the electrolyte thin-film layer coating the catalyst), *w*_CO_2_(g)_ is the mass fraction of CO_2(g)_, *D*_CO_2_(aq)_ is the diffusion coefficient for CO_2(aq)_ in water, *H*_CO_2_(aq)_ is Henrys constant for CO_2_ in water at 298 K, *p*_g_ is the gas pressure, *a*_c_ is the specific surface area of the cathode, and *C*_CO_2_(aq)_ is the concentration of CO_2(aq)_. For CO_2(aq)_ and CO_2(g)_, solvation (eq 8) also contributes to the rate of production/consumption. Solvation between phases is simulated only for CO_2_.

### Equations Governing Transport of Species and Electrons—Mass Balance and Current Balance

Mass balance for species in the solution phase is governed by the Nernst–Planck equation:
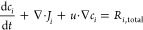
9where *u* is the convective velocity (which is zero in this simulation), *R*_*i*,total_ is the total rate of production/consumption of species *i* due to EC, solution-phase reactions, and solvation reactions:
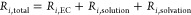
10

The molar flux *J*_*i*_ of each species is defined according to the Nernst–Planck equation, describing transport due to diffusion and migration at infinite dilution:
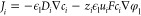
11where ϵ_l_ is the porosity of the given domain, *D*_*i*_ is the diffusion coefficient for species *i* in solution, *z*_*i*_ is its charge, and *u*_*i*_ is its mobility according to the Nernst–Einstein relation. Increasing the porosity will allow for increased transport of liquid species but result in a decrease in the current/transport of electrons (eq 14 below) and of gases. The solid volume fraction ϵ_s_ is calculated from the porosity as (ϵ_s_ = 1 – ϵ_l_). In the cathode, the liquid volume fraction is (ϵ_l_*S*), and the gas volume fraction is (ϵ_l_(1 – *S*)), where the saturation *S* = 0.64 as in Weng et al.^9^ This results in the anode and membrane being 60% liquid and 40% solid and the cathode being 39% liquid, 21% gas, and 40% solid.

Electrons are not treated as a chemical species with mass; their transport is governed by the current balance in the solid phase. Current balance is governed by eqs 12 and 13:
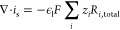
12
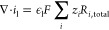
13where *i*_s_ and *i*_l_ are the current densities in the solid and solution (liquid) phases, respectively, given by
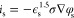
14and
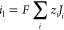
15where σ is the conductivity of the solid phase (i.e., the electrode), and φ_s_ is the potential in the solid phase. Mass transport of gas-phase species *n* is governed by
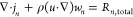
16where *ρ* is the mixture density, *u* is the average velocity of the mixture, and *w*_*n*_ is the mass fraction of species *n*, where we impose the condition ∑_*n*_*w*_*n*_ = 1. The mass flux *j*_*n*_ is defined as
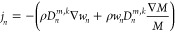
17where *M* is the mean molar mass . The diffusion coefficient *D*_*n*_^*m*,*k*^ is calculated as , where the Stefan–Maxwell diffusivity  and the Knudsen diffusivity  are occurring in parallel. Here, *x*_*n*_ is the mole fraction of species *n*. Darcy’s law governs the convective velocity according to
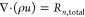
18

In eqs 16 and 18, *R*_*n*,total_ for gas-phase species is defined by eq 7 for CO_2_, and it is zero for other gaseous species. The convective velocity, μ, is defined as
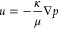
19where *p* is the pressure, κ is the permeability of the medium (calculated from the Kozeny–Carman relation where the particle diameter is taken as twice the pore radius), and μ is the dynamic viscosity of the mixture. Electroneutrality is assumed in all domains of the simulation:
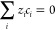
20

### Boundary Conditions

At the leftmost boundary (gas channel/cathode boundary), the electric potential of the solid phase is fixed (φ_s_ = *E*_applied_), and at the rightmost boundary (anode/anolyte channel boundary) it is set to ground (φ_s_ = 0 V), corresponding to the locations of the current collectors.

At the leftmost boundary, the mole fraction of CO_2(g)_ is fixed corresponding to the inlet feed composition; 99% for CO_2(g)_, <1% for CO_(g)_/H_2(g)_. At this boundary, the fluxes of gaseous species can be measured, allowing us to estimate the composition of the gas channel. The flux of CO_2(g)_ is set to  at the cathode/membrane boundary, where *r*_9_ is the rate of reaction R9, CO_2(g)_ ⇄ CO_2(aq)_. The fluxes of other gaseous species are set to zero at this boundary.

At the anode/anolyte channel boundary, the concentrations of solution-phase species H_2_O_(aq)_, H_(aq)_^+^, OH_(aq)_^–^, HCO_3(aq)_^–^, CO_3(aq)_^2–^, and K_(aq)_^+^ are fixed at their equilibrium concentrations corresponding to the delivery of anolyte, either 0.1 M KHCO_3_ or 0.1 M KOH. These values are given in the Supporting Information.

### Input Parameters

The equations above are populated with parameters from Table 2 before being solved.

When solving the simulation in COMSOL, a user-controlled mesh was used with 1000 mesh elements in the anode and cathode domains combined, and 100 in the membrane domain. One mesh refinement was performed in the membrane domain. The solution was independent of an increasing number of mesh elements. The default PARADISO solver was used. Separate study steps were used to increase the membrane fixed charge to its final value and to increase the voltage from 1 to 3 V. For each step, the maximum number of iterations was increased to 1000, and the relative tolerance was 1 × 10^–4^.

### Sensitivity Analysis Methodology

First, a base case simulation was defined as outlined above and solved. The simulation was then solved again once for each parameter of interest, where the parameter was increased by 1% from its original value. The sensitivity, *s*, of the CO partial current density, *i*_CO_, to each parameter is defined as
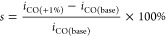
where *i*_CO(+1%)_ is the CO partial current density of the simulation with the parameter increased by 1%, and *i*_CO(base)_ is the CO partial current density of the base case simulation. Sensitivity analysis was also performed for a 0.1% perturbation of the input parameters, and the same sensitivity trends are reported (Figure S5 of the Supporting Information).

## Results and Discussion

### Polarization Curve and Faradaic Efficiency

**Figure 2:**
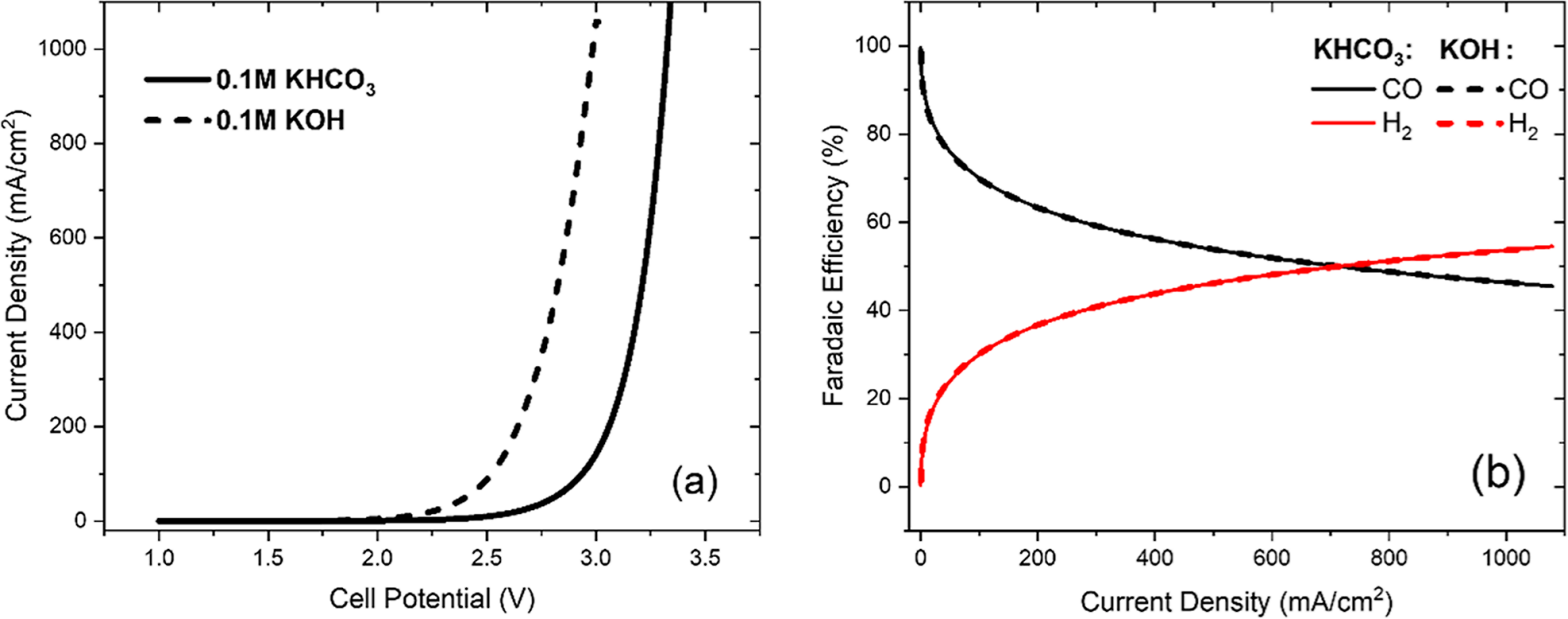
(a) Total current density vs the applied cell potential and (b) Faradaic efficiencies of each product vs current density, for the 0.1 M KHCO_3_ and 0.1 M KOH cases. The solid line represents the KHCO_3_ case and the dashed line the KOH case.

Figure 2a shows that using KOH as the electrolyte instead of KHCO_3_ will result in a greater current density at the same potential as expected.^11,36^ This is due to a reduction in the Nernstian (concentration) overpotential at the anode, due to the constant supply of OH^–^ via the anolyte from KOH.^11^ OH^–^ also has a greater ionic conductivity than HCO_3_^–^, resulting in less Ohmic resistance in the cell.

The FE is a measure of the utilization efficiency of the electrons in the system—it is defined as the fraction of the electrons, which go to the formation of the relevant product (CO vs H_2_). The FE of CO is calculated as . Figure 2b shows that at higher current densities, H_2(g)_ formation dominates over CO_(g)_ formation at the cathode, as in the experiment of Larrazabal et al.^37^ No significant difference is observed in the simulation for the FE of the KHCO_3_ and the KOH cases at the same current densities;^11^ a difference is only observed when plotting FE vs potential, since each case gives a different polarization curve. We note that the cation type and concentration are reported to affect the activity and selectivity of EC reactions, although the mechanism through which this occurs is not fully understood. One possible explanation is through the stabilization of reaction intermediates.^38^ With the current state of art in our knowledge of these processes, multiphysics simulations such as those presented here do not account for reaction intermediates, as discussed later, or a full set of solution-phase reactions including those involving K^+^. We further note that a range of different experimental Faradaic efficiencies have been reported in the literature for GDE electrolysis cells, and the EC parameters used here replicate the behaviors reported by Larrazabal et al.^37^ whose setup this simulation resembles.

### Concentrations

**Figure 3:**
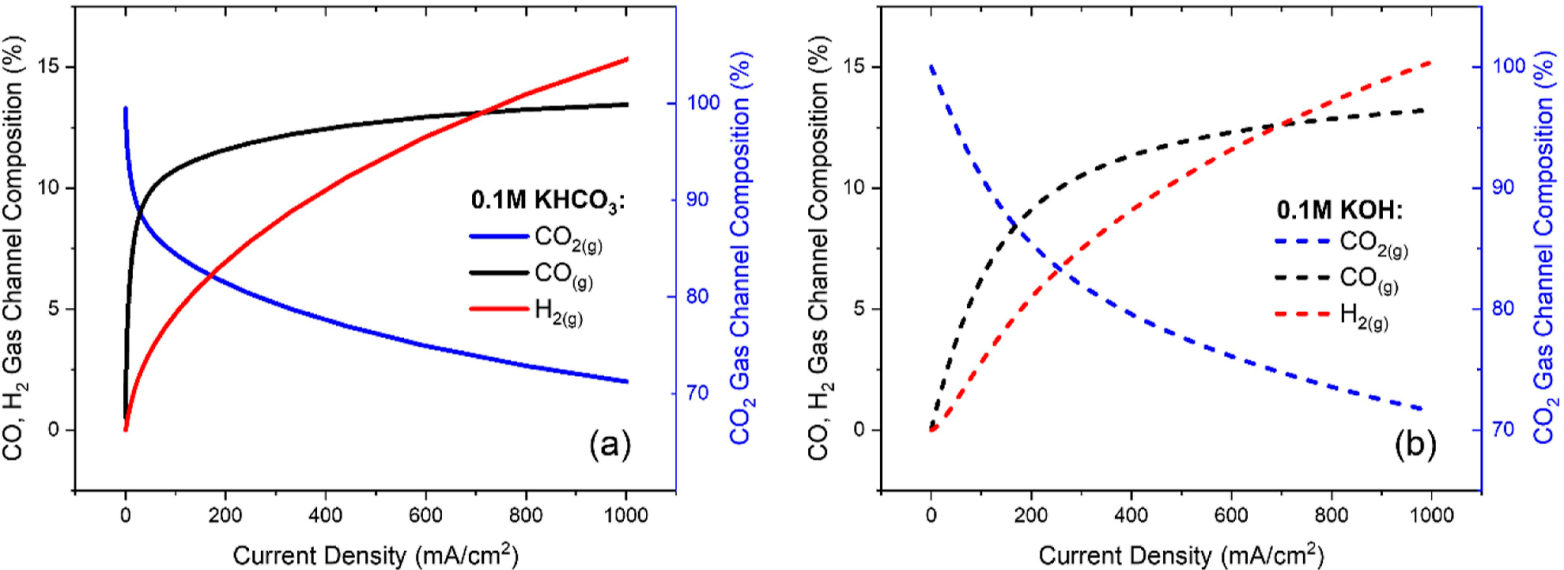
Estimated percentages of species CO_2(g)_, CO_(g)_, and H_2(g)_ in the gas channel vs current density, for (a) 0.1 M KHCO_3_ and (b) 0.1 M KOH. The gas channel composition is estimated by measuring the fluxes of each species at the gas channel/cathode boundary.

Figure 3a,b shows the percentages of CO_2__(g)_, CO_(g)_, and H_2__(g)_ in the gas channel for the 0.1 M KHCO_3_ and 0.1 M KOH cases. The composition is estimated by measuring the molar flux of each species at the cathode/gas channel boundary and dividing by the total flux of all species (CO_2__(g)_, CO_(g)_, and H_2__(g)_) at that boundary. In the simulation, the mole fractions are fixed at the left-hand boundary at the inlet feed composition, which is 99% CO_2__(g)_ and <1% CO_(g)_/H_2__(g)_ (i.e., the simulation assumes a sufficiently high flow rate of CO_2__(g)_ to maintain this composition). With applied current density, the concentrations of CO_(g)_ and H_2__(g)_ in the gas channel increase while the concentration of CO_2__(g)_ decreases. For the KOH case, the CO_2__(aq)_ being consumed at the anode in the solution phase results in diffusion out of the cathode into the membrane, and thus a lower CO_2_ utilization at very low current densities is observed compared to the KHCO_3_ case (see Table 3).

**Table 3:** Utilization of CO_2(aq)_ for the KHCO_3_ and KOH Cases, Corresponding to the Dashed Region in Figure 5^a^

current density (mA/cm^2^)	ε_CO_: CO_2__(aq)_ converted to CO_(g)_, (%)	ε_solution_: CO_2__(aq)_ converted to HCO_3_^–^_(aq)_ or CO_3_^2–^_(aq)_ (%)	ε_diffusion_: CO_2__(aq)_ diffuses from cathode (%)
**0.1 M KHCO_3_ Case**			
**10**	52% (8.1×10^–4^)	37% (5.7×10^–4^)	11% (1.8×10^–4^)
**100**	53% (6.9×10^–3^)	41% (5.2×10^–3^)	6%(7.8×10^–4^)
**1000**	47% (5.2×10^–2^)	50% (5.6×10^–2^)	3% (3.3×10^–3^)
**0.1 M KOH Case**			
**10**	28% (1.1×10^–3^)	25% (8.8×10^–4^)	47% (1.6×10^–3^)
**100**	51% (8.1×10^–3^)	38% (6.0 × 10^–3^)	11% (1.5× 10^–3^)
**1000**	47% (4.4×10^–2^)	50% (4.6×10^–2^)	3% (3.0×10–3)

a For both cases, utilization (ε_CO_) converges to ∼50% at higher current densities.^11^ The magnitudes of each process in mol/m^2^/s are included in parentheses for reference.

**Figure 4:**
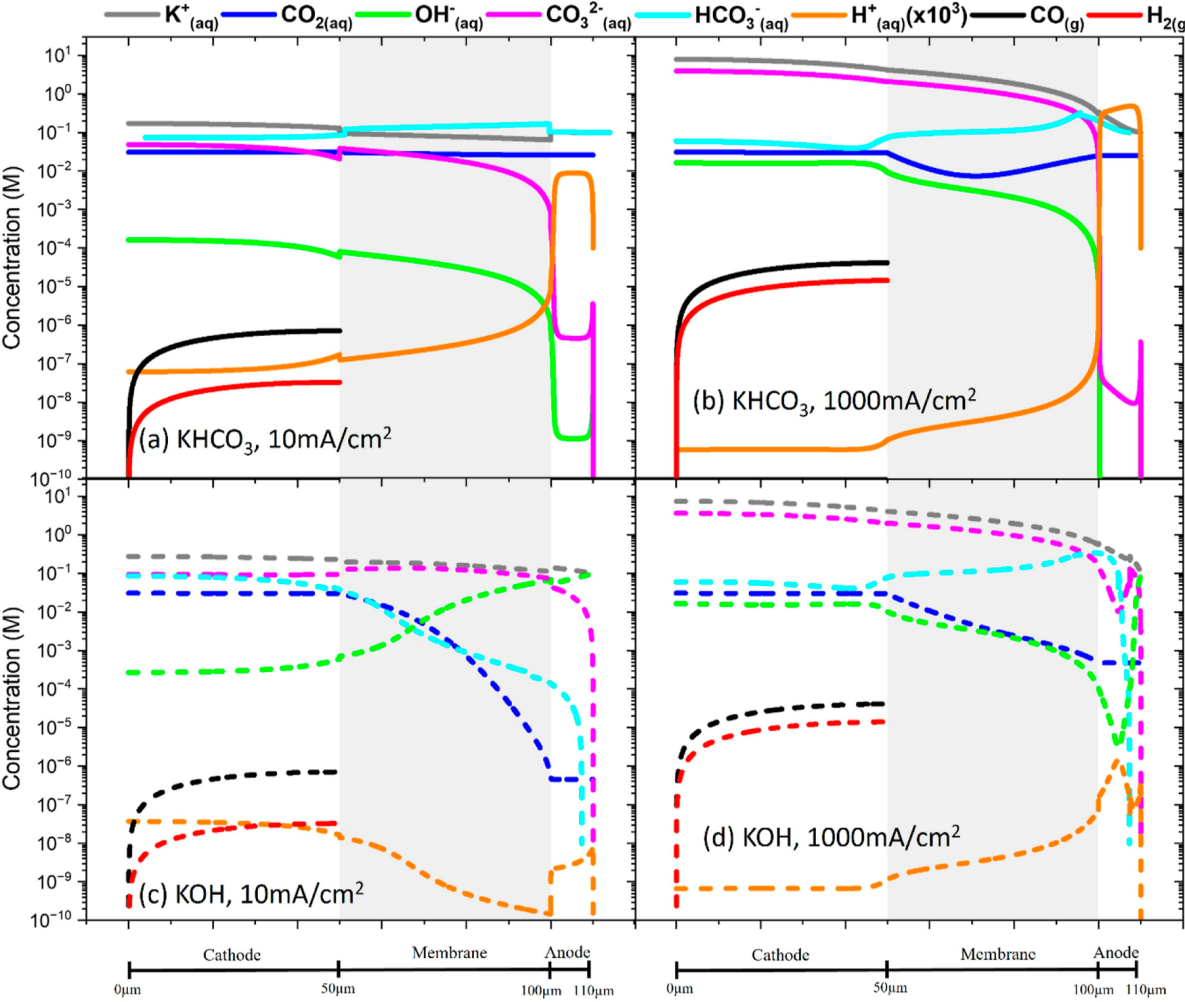
Concentrations of all species in all domains of the cell. The solid lines represent the 0.1 M KHCO_3_ case and the dashed lines the 0.1 M KOH case: (a) KHCO_3_ 10 mA/cm^2^, (b) KHCO_3_ 1000 mA/cm^2^, (c) KOH 10 mA/cm^2^, and (d) KOH 1000 mA/cm^2^. CO_2(aq)_ forms in the solution phase from CO_2(g)_ within the cathode. Concentrations of H_2_O_(aq)_, H_(aq)_^+^, OH_(aq)_^–^, HCO_3(aq)_^–^, CO_3(aq)_^2–^, and K_(aq)_^+^ are fixed at the rightmost boundary corresponding to the feed of the anolyte solution in each case. Note that the H_(aq)_^+^ concentration is multiplied by 10^3^, so it appears higher in the figure.

Figure 4a–d shows the concentration profiles of all species in all domains of the cell, for both the 0.1 M KHCO_3_ and 0.1 M KOH cases, at 10 and 1000 mA/cm^2^ showing the effects of applied current density and electrolyte type. CO_2(aq)_ is formed from CO_2(g)_ within the cathode, where its concentration is maintained at ∼30 mM. For the KHCO_3_ case, Figure 4a,b, there is always a relatively high amount of CO_2(aq)_ at the anode, as HCO_3(aq)_^–^ is supplied via the anolyte, which is converted back to CO_2(aq)_ via reaction R4. At the anode, CO_2(aq)_ exits via dissolution to CO_2(g)_. At higher current densities, CO_2(aq)_ in the membrane is also consumed by OH_(aq)_^–^, which is produced during CO_2_ reduction at the cathode. For the KOH case, Figure 4c,d, we are supplying OH_(aq)_^–^ via the anolyte, which consumes CO_2(aq)_ in the anode region via reaction R6. Thus, there is less CO_2(aq)_ at the anode, compared to the KHCO_3_ case. This net consumption of KOH and conversion to (bi)carbonates is described by Rabinowitz and Kanan^24^ and identified as a problem in electrolyzers of this type. For the KOH case, the anode contains more CO_2(aq)_ at high current densities as HCO_3(aq)_^–^ combines with H_(aq)_^+^ via reaction R4.

The HCO_3(aq)_^–^ profile shown in Figure 4a,b for the KHCO_3_ case shows the HCO_3__(aq)_^–^ concentration fixed at 0.1 M at the anode corresponding to 0.1 M KHCO_3_ electrolyte used. HCO_3(aq)_^–^ is produced at the cathode via reaction R6 and subsequently is converted to CO_3(aq)_^2–^ via reaction R7. High concentrations of CO_3(aq)_^2–^ and of K_(aq)_^+^ at the cathode at higher potentials indicates salt (K_2_CO_3_/KHCO_3_) formation. For the KOH case, the concentration of HCO_3(aq)_^–^ is higher at the anode at higher current densities due to the conversion of H_(aq)_^+^ to HCO_3(aq)_^–^. The concentrations of all ions are shifted in the AEM depending on their charge, as anions are attracted to and cations repelled from the membrane.

OH_(aq)_^–^ is generated at the cathode electrochemically via CO_2_ reduction and is consumed at the anode electrochemically by reaction R3b. For the KOH case, the concentration of OH_(aq)_^–^ is fixed at the anode boundary at 0.1 M, corresponding to a fresh supply of KOH. OH_(aq)_^–^ is consumed by reaction R6, which is responsible for producing HCO_3(aq)_^–^.^24^

K_(aq)_^+^ is fixed at 0.1 M at the anode for both cases. K_(aq)_^+^ migrates toward the cathode due to its negative charge and due to the high presence of anions there (HCO_3(aq)_^–^, and CO_3(aq)_^2–^). It is important to note that K_(aq)_^+^ is not involved in any chemical reactions in this simulation. This is a simplification that could be amended by including a full set of reactions, as discussed in the Supporting Information. High concentrations of K_(aq)_^+^ at the cathode at higher potentials indicate salt formation (the solubility limit is ∼8 M for K_2_CO_3_). This occurs at approximately 750 mA/cm^2^, according to Weng et al.^11^

CO_3(aq)_^2–^ is fixed at the anode boundary at its equilibrium value for each case. CO_3(aq)_^2–^ is produced at the cathode region primarily via reaction R7 due to the presence of OH^–^ ions. As is the case for K_(aq)_^+^, a high concentration of CO_3(aq)_^2–^ at the cathode at higher current densities indicates salt formation.

H_(aq)_^+^ is fixed at the anode boundary at its equilibrium value for each case. The EC oxidation of water (reaction R3a) results in a higher H_(aq)_^+^ concentration at the anode. The concentration of H_(aq)_^+^ is low at the cathode where OH_(aq)_^–^ is produced electrochemically. Note that the H_(aq)_^+^ concentration is multiplied by 10^3^ so it appears higher in the figure.

The concentrations of gaseous species CO_(g)_ and H_2(g)_ are also shown. Although H_2(g)_ is produced at a higher rate at 1000 mA/cm^2^ than CO_(g)_, the CO_(g)_ concentration is higher since H_2(g)_ diffuses out of the cathode more easily, hence why we estimate the gas channel composition as in Figure 3a,b rather than paying particular attention to the gaseous concentrations within the cathode. We also note that the volume fractions in the solution phase and gas phase are different because of the porosity values used here, as discussed in the Methodology section.

### CO_2_ Utilization

**Figure 5:**
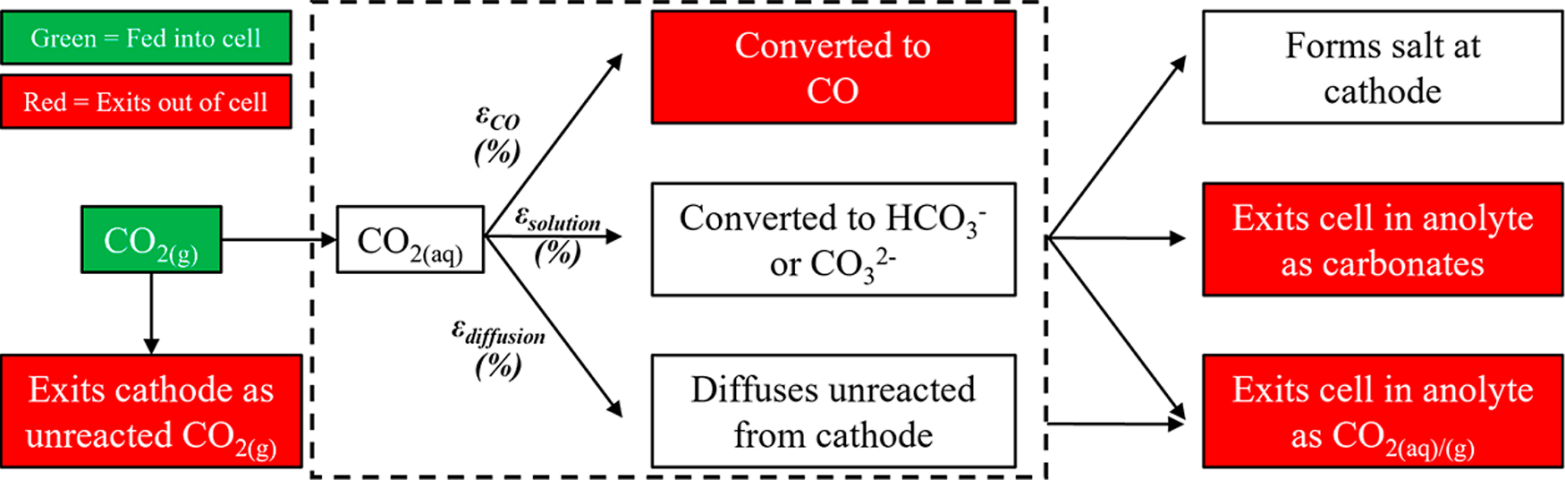
Flux diagram for CO_2_ in the zero-gap MEA cell. The region inside the dashed border represents the processes analyzed using the 1D simulation. A key problem for this setup is competition between the EC reduction of CO_2(aq)_ to CO_(g)_ and solution-phase reactions converting CO_2(aq)_ to HCO_3(aq)_^–^/CO_3(aq)_^2–^.

As well as current density and FE, CO_2_ utilization is an important metric in determining the performance of CO_2_ electrolysis cells. CO_2_ utilization is a key problem for zero-gap MEA cells, as there is competition between the EC reduction of CO_2(aq)_ and solution-phase reactions converting CO_2(aq)_ to HCO_3_^–^/CO_3_^2–^.^4,13,24^Figure 5 shows a mass flux diagram for CO_2_ in a typical MEA-type cell, illustrating this competition. Table 3 indicates the rates of each process ε in Figure 5; CO_2(aq)_ to CO_(g)_, CO_2(aq)_ consumed into solution phase (converted to HCO_3_^–^/CO_3_^2–^), and CO_2(aq)_ diffusing unreacted out of the cathode to the anode side. Utilization here is calculated as
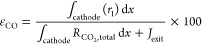
where *r*_1_ refers to the reaction rate of R1 (i.e., CO_2(aq)_ reduced to CO), *R*_CO_2_,total_ is the total rate of CO_2_ consumed, and *J*_exit_ is the flux of CO_2_ exiting the cathode. To find the rate of CO_2(aq)_ consumed, *R*_CO_2_,total_, we sum the rates of reactions R1, R4, and R6. The conversions ε_solution_ and ε_diffusion_ are calculated similarly. The integrals are computed over the length of the cathode.

At low current densities for the KOH case, the concentration gradient of CO_2(aq)_ between the cathode and anode means a high rate of diffusion of CO_2(aq)_ from the cathode, thus lowering both the fraction of CO_2(aq)_ converted to CO (ε_CO_) as well as the fraction of CO_2(aq)_ consumed in solution in the cathode (ε_solution_). We can see that for the KHCO_3_ and KOH cases, the CO_2(aq)_ utilization ε_CO_ converges toward ∼50% at higher current densities, while diffusion out of the cathode becomes negligible. As discussed in Weng et al.^11^ and Liu et al.,^39^ studying the reaction stoichiometry suggests a utilization of ∼50% for these reactors (depending on the relative rates of each reaction). This upper limit is imposed by the zero-gap nature of the cells, resulting in competition between the solution-phase and EC reactions. As in the simulation of Weng et al.,^11^ high CO_2_ utilization can be achieved at relatively low current densities of approximately 100 mA/cm^2^. This is advisable to prevent salting, flooding, and wear of materials, which all occur at higher current densities. Parameters of the multiphysics simulation can be tuned to find the optimal conditions under which to run the cell, but that is outside the scope of this paper.

### Sensitivity Analysis

**Figure 6:**
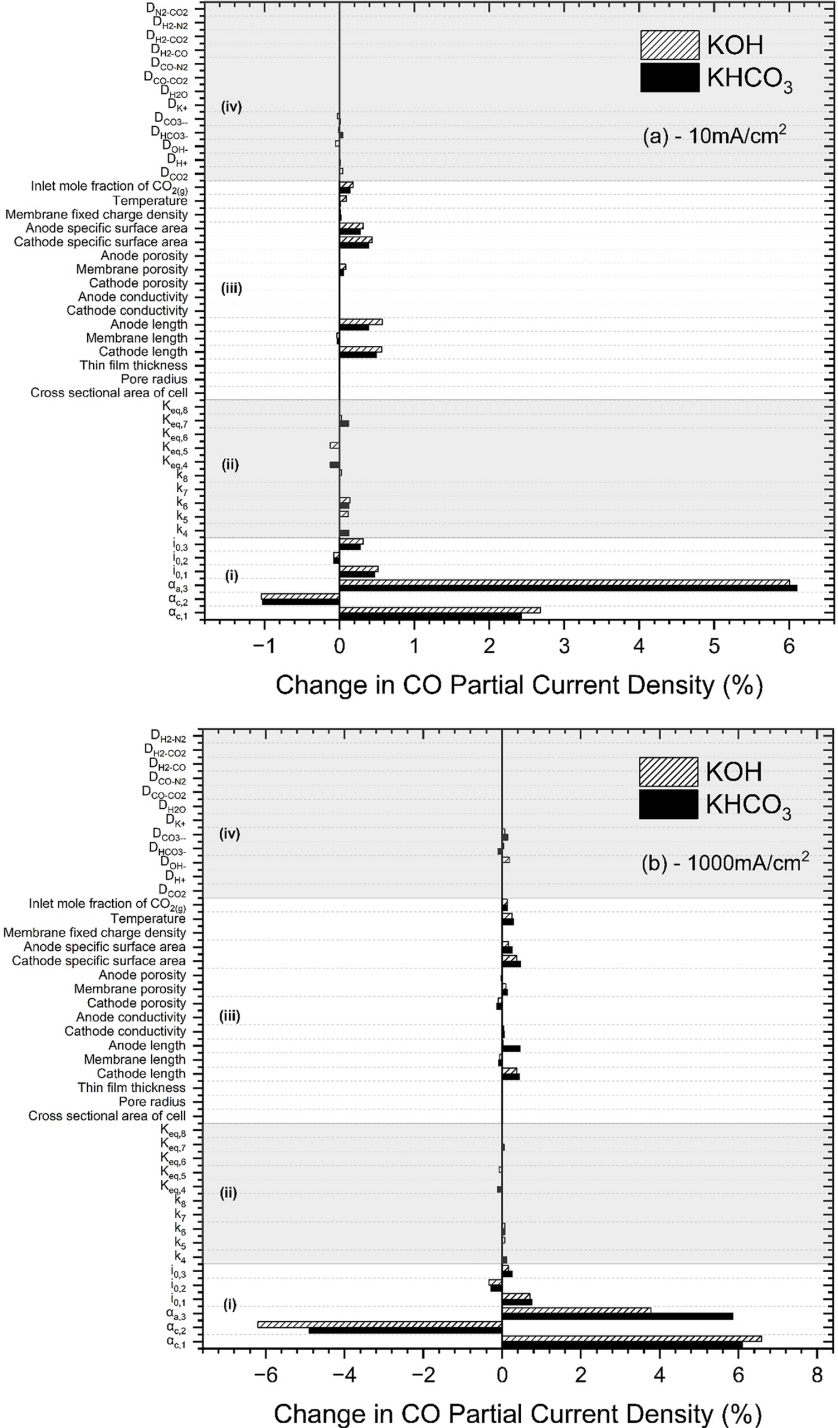
Sensitivity of the multiphysics simulation to each input parameter for the KOH and KHCO_3_ cases (a) at 10 mA/cm^2^ (b) at 1000 mA/cm^2^. The sensitivity is defined as the percentage change in the partial current density of CO (*i*_CO_) with a 1% increase in the given parameter. Parameters are divided into categories (i) EC kinetic parameters, (ii) solution-phase kinetic parameters, (iii) physical parameters, and (iv) diffusion coefficients.

The results of sensitivity analysis are shown in Figure 6a,b for the 0.1 M KOH and 0.1 M KHCO_3_ cases, at 10 and 1000 mA/cm^2^. We can see that, at any condition, *i*_CO_ is highly sensitive to the kinetic parameters (*i*_0_, α) of all EC reactions. Even at high currents, i.e., in the “mass transport-controlled regime”, these parameters give the biggest change in *i*_CO_ (although diffusion coefficients show a higher response at higher current densities as expected). We can thus conclude that to make the greatest gains in *i*_CO_, effort should be focused toward finding better-performing catalysts with high activity toward the desired product. Indeed, this is where most work is done today in CO_2_ reduction.^40^ The higher sensitivity to kinetic parameters of reaction R2, the hydrogen evolution reaction, is due to this reaction dominating over CO_2_ reduction at high current densities.

It is important to note that the simulation is also highly sensitive to the parameters of reactions R3a and R3b, which are conventionally called the OER parameters. Whether the simulation models the OER or not and the choice of OER parameters will affect the extracted *i*_0_/α_c_ for reaction R1 since many simulations exclude the anode. We also note that it is common to round these parameters, as well as kinetic parameters of solution-phase reactions, to the nearest order of magnitude from their reference source—doing so will impact the simulation results significantly as well as the extracted *i*_0_/α_c_ values.

*i*_CO_ also shows high sensitivity to physical parameters, e.g., the lengths and specific surface areas of the electrodes. High sensitivity of the simulation to these physical parameters indicates the need to ensure that the simulated conditions precisely match those of the experiment if one is using a multiphysics simulation to make accurate predictions about one’s experiment. Further, if using a multiphysics simulation together with an experimental setup to extract EC kinetic parameters, then incorrect values will be extracted if the parameters such as lengths of simulation and experiment are not precisely matching.

Lower sensitivity is shown to rate constants for solution-phase reactions and for diffusion coefficients. However, this does not imply that these parameters are not important for the simulation, only that they do not impact *i*_CO_ in particular. Other metrics of interest may be highly sensitive to these parameters. Given the scale of the uncertainty in solution-phase kinetic parameters, in particular, we recommend that further efforts are made to determine their values more precisely.

Since many parameters are difficult to determine, and simulation output is highly sensitive to many parameters, it is likely that such multiphysics simulations will not be able to make precise quantitative predictions about cell performance, e.g., predicting exact species concentrations. We recommend therefore that simulations of this kind, working with high levels of uncertainty in their input parameters, should be used to investigate trends rather than to make precise quantitative predictions, unless parameters are determined more precisely. We further note that infidelity in the simulation due to inaccurate input parameters will propagate the nature of the highly coupled reaction network in this system (Figure S6).

A sensitivity analysis of this nature can also help in determining which parameters will affect the convergence of these simulations the most if convergence issues arise due to unphysical quantities being computed. Convergence is an issue limiting the development of these models.^4,15,17,23,27^ By choosing a different sensitivity metric other than *i*_CO_, one could optimize cell performance by identifying which parameter most affects the metric of interest, e.g., FE or CO_2_ utilization.

**Figure 7:**
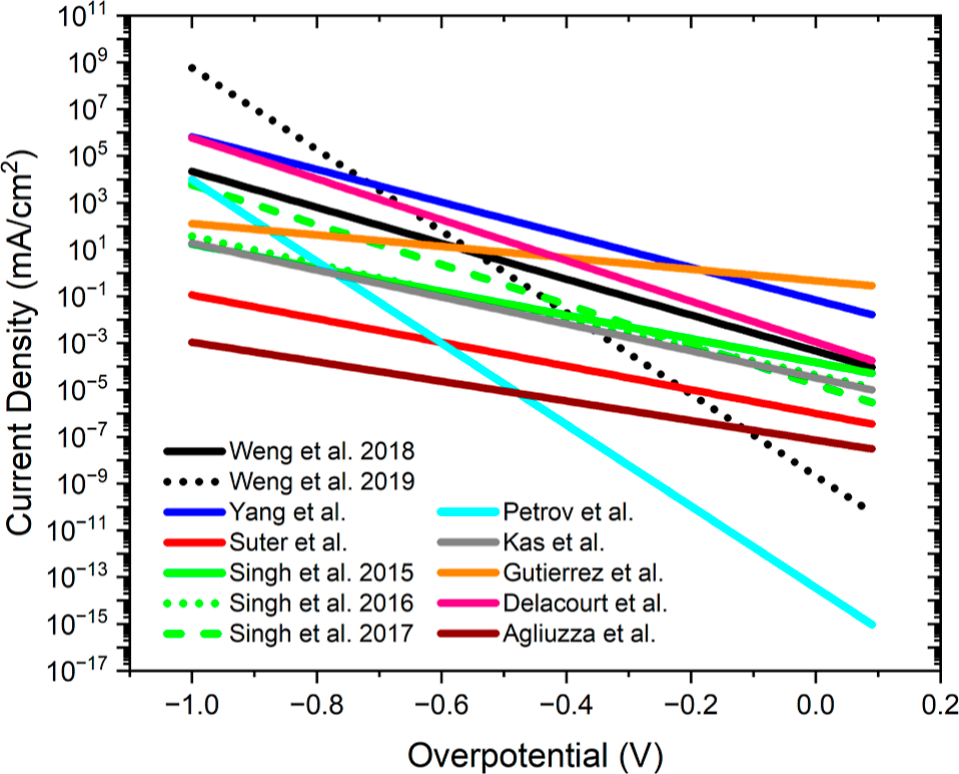
Reported current densities of reaction R1 (CO_2(aq)_ + H_2_O_(aq)_ + 2e^–^ → CO_(g)_ + 2OH_(aq)_^–^) vs overpotential, using the *i*_0,1_/α_c,1_ pairs given in Table 1 from the literature.^6−9,11,17,19,21−23,28,30^ Several orders of magnitude variance is observed between these simulated current densities. The figure is generated by plotting the Tafel equation (eq 1) versus overpotential in each case.

The Tafel equation is plotted in Figure 7 showing the current density of reaction R1 (CO_2_ reduction to CO) vs overpotential for each of the *i*_0,1_/α_c,1_ pairs reported in Table 1. In the Tafel equation, *i*_0_ (exchange current density) represents the current density at a 0 V overpotential, and α (kinetic transfer coefficient) represents the sensitivity of the reaction rate to the overpotential.

We can see that each *i*_0,1_/α_c,1_ pair results in significantly different behaviors. Some disagreement in the reported values of *i*_0,1_/α_c,1_ should be expected, however, since kinetics will be affected not only by the catalyst material but also by electrolyte concentration, cation type, surface coverage, partial pressure of CO_2_, and morphology of the catalyst.^8,41,42^ The Tafel slope (or equivalently α) is also reported to change vs overpotential.^41^ Multiphysics simulations generally do not account for the fact that *i*_0_/α changes with conditions but use one number for *i*_0_/α in all conditions. This is another reason to expect infidelity in such simulations vs experiment. Table 1 shows that significantly different conditions are simulated in each instance. Thus, we should not expect just one *i*_0_/α pair for these simulations for any reaction.

Another important consideration, overlooked in multiphysics simulations, is that the CO_2_ reduction reaction is modeled as a single step, when in reality it is multiple steps, each step having its own rate, and is condition dependent.^41^ Multiphysics simulations model this reaction as a single step using the *i*_0_/α of the rate-determining step. Which step is the rate-determining step, however, will change with the condition, hence using a single *i*_0,1_/α_c,1_ pair for the CO_2_ reduction reaction under all conditions will result in infidelity. To accurately extract kinetic parameters *i*_0_/α using multiphysics simulations, one should account for the multistep nature of EC reactions, as well as condition-dependent kinetics, which is often overlooked. Attempts have been made to determine *i*_0,1_/α_c,1_ more rigorously^8,41,42^ by combining multiphysics models for kinetics and transport with quantum mechanical models using density functional theory (DFT). These works account for the multiple steps in each EC reaction mechanism and how their rate constants change in different conditions. Combining a DFT model with a microkinetic and transport model will more accurately reproduce experimental results from CO_2_ electrolysis cells. Singh^8^ et al. use a combined multiphysics and DFT model to determine *i*_0,1_/α_c,1_ for their specific Ag catalyst in question. A multiphysics simulation alone can still be used to gain insights into cell behaviors, which are due to reaction/transport phenomena, but to build the simulation such *i*_0_/α needs to be chosen which accurately replicates cell behavior.

## Conclusions

A zero-gap membrane electrode assembly cell for CO_2_ reduction is simulated in 1D, considering EC and solution-phase reactions and species transport due to diffusion and migration under a zero-flow condition. The cathode is a porous Ag GDE, and the anode is a porous iridium oxide electrode supplied with aqueous 0.1 M KOH/KHCO_3_. It is shown that a significant difference in the polarization curve is observed between the 0.1 M KHCO_3_ and 0.1 M KOH cases, mostly due to the constant supply of OH_(aq)_^–^ at the anode for the KOH case, resulting in a more favorable anodic overpotential. At the cathode, CO formation dominates at lower current densities and is surpassed by H_2_ production at current densities higher than ∼700 mA/cm^2^, which is in agreement with observed experimental trends for electrolyzers of this type. Flux analysis shows that CO_2(aq)_ is consumed by three competitive processes; conversion to CO, conversion to carbonates (HCO_3_^–^/CO_3_^2–^), and diffusion out of the cathode. In agreement with previous experimental work, the CO_2_ utilization metric shows that CO_2_ reduction to CO is limited to ∼50% due to competition between EC reduction of CO_2(aq)_ and its solution-phase reaction with OH_(aq)_^–^ to form the carbonates. Furthermore, if KOH is used as the electrolyte, KOH will be consumed by solution-phase reactions at the anode, forming carbonates.

Sensitivity analysis of the multiphysics simulation shows that the current density of the CO_2_ reduction reaction (*i*_CO_) is highly sensitive to the EC kinetic parameters of all EC reactions occurring: (i) CO_2_R, (ii) HER, and (iii) OER. For the CO_2_R reaction, it is further shown that the range of EC kinetic parameters *i*_0_ and α suggested in the literature for Ag electrodes results in variance in partial current densities spanning 12 orders of magnitude. The sensitivity of *i*_CO_ to many of the simulation’s input parameters helps to explain the large range in the reported values of *i*_0_ and α, which have been extracted by multiphysics simulations fitting *i*_CO_ to experimental data. It is thus concluded that the veracity of multiphysics simulations is dependent on improved knowledge of the EC reactions taking place and of their chemical kinetics. However, it is noted that multiphysics simulations still offer utility if appropriately informed by experiment. Finally, the sensitivity analysis methodology presented here can also be employed to optimize cell performance, by giving the most important parameter with respect to any performance metric such as *i*_CO_, FE, energy efficiency, CO_2_ utilization, etc.

## Supporting Information Available

Sensitivity analysis raw data; results of sensitivity analysis performed for 0.1% perturbation; plot of *i*_0_/α_c,CO_ pairs found in literature; discussion on input parameters as sources of infidelity in multiphysics simulations; concentrations of aqueous species used in the simulation; and possible chemical reactions which are not simulated in CO_2_ reduction simulations (PDF)

## List of Terms

*i*: current density
*i*_0_: exchange current density
α: kinetic transfer coefficient
η: overpotential
*A*: Tafel slope
ρ_mem_: membrane fixed charge
*E*_0_: standard reduction potential
ϵ: porosity
*L*: length
*R*_*i*/*n*_: rate of production/consumption of a species *i* or *n*
*a*: specific surface area
*v*: stoichiometric coefficient
*k*: rate constant for solution-phase reaction
*K*_eq_: equilibrium constant for solution-phase reaction
*r*: rate of reaction
*k*_GL_: gas to liquid mass transfer coefficient
*p*_g_: gas pressure
*w*: mass fraction
*c*: concentration
*D*: diffusion coefficient
δ_TF_: thin-film thickness of electrolyte layer on electrode surface
*u*: convective velocity
*J*: molar flux
*z*: charge
*u*: mobility
φ: potential
σ: conductivity
*j*: mass flux
ρ: density
*M*: mean molar mass
*r*_p_: pore radius
*x*: mole fraction
*k*: permeability
μ: dynamic viscosity

## Superscripts

m,k: Maxwell and Knudsen diffusion
r: reverse

## Subscripts

solution: solution-phase reaction
EC: electrochemical/surface reaction
l: liquid phase
s: solid phase
*n*: gas-phase species
*i*: solution-phase species
*m*: electrochemical/surface reaction
*j*: solution-phase reaction
c/a: cathodic/anodic
