# Supplementary material for: Sensitivity Analysis of One-Dimensional Multiphysics Simulation of CO2 Electrolysis Cell
Source: J Phys Chem C Nanomater Interfaces. 2024 Jun 27;128(27):11131–44. doi: 10.1021/acs.jpcc.4c00690 (PMC11247494; doi:10.1021/acs.jpcc.4c00690)
Supplement: Supplementary file 1 — jp4c00690_si_001.pdf [file jp4c00690_si_001.pdf]

Supporting Information: 'Sensitivity Analysis of One-Dimensional Multiphysics Simulation of CO_2 Electrolysis Cell'

Harry Dunne[*1], *Weiming Liu*[1], *Mohammad Reza Ghaani*[1], *Kim McKelvey*[2], *Stephen Dooley*[1]

[1]School of Physics, Trinity College Dublin, Ireland

[2] MacDiarmid Institute for Advanced Materials and Nanotechnology and School of Chemical and Physical Sciences, Victoria University of Wellington, Wellington 6012, New Zealand

Contents

i_0/α Pairs in Literature:....................3

Input Parameters as Sources of Infidelity in Multiphysics Simulations:....................6

Sensitivity Analysis Data:....................8

Sensitivity Analysis Performed with 0.01% Perturbation:....................12

Reactions Not Considered in Multiphysics Simulations for CO_2 Reduction:....................13

Values of Aqueous Species in Anolyte Solution:....................14

Discussion On Error Propagation Due to Network of Reactions:....................15

References:....................16

i0/α Pairs in Literature:

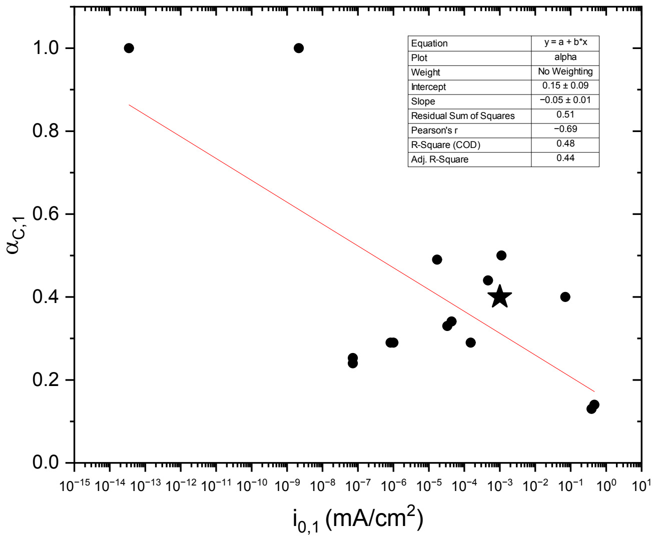

Figure S1: plot of i_0/α pairs from Table 1, for the COER reaction. As shown in Bui [1] et al, there is a weak correlation between $i_{0,1}$ and $\alpha_{c,1}$ which implies that several equally good fits are possible. The star indicates the value used in this work.

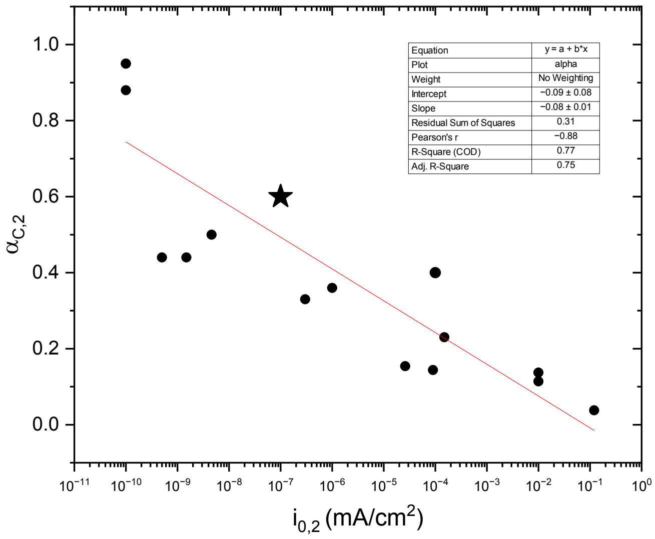

Figure S2: plot of i_0/α pairs for Reaction 2 (from the simulations in Table 1 who include this reaction). As shown in Bui [1] et al, there is a correlation between $i_{0,2}$ and $\alpha_{c,2}$ *which implies that more several equally good fits are possible. The star indicates the value used in this work.*

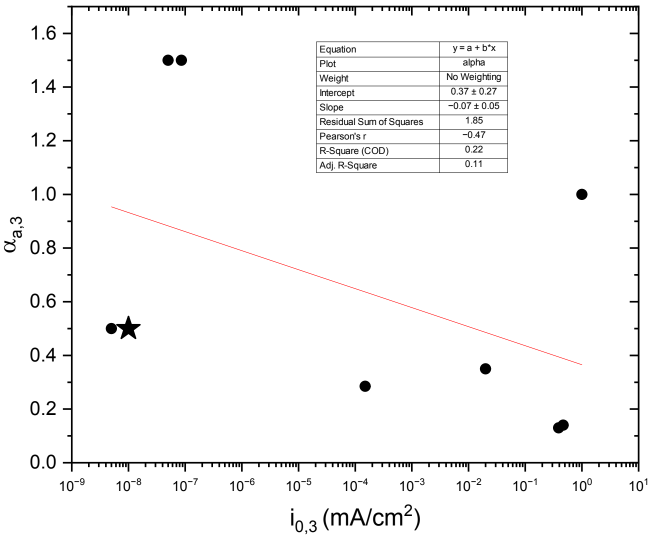

Figure S3: plot of i_0/α pairs for Reaction 3 (from the simulations in Table 1 who include this reaction). No correlation should be expected here since these studies do not all use the same catalyst at the anode. The star indicates the value used in this work. Here $\alpha_{a,3}$ refers to the anodic transfer coefficient in the Tafel equation.

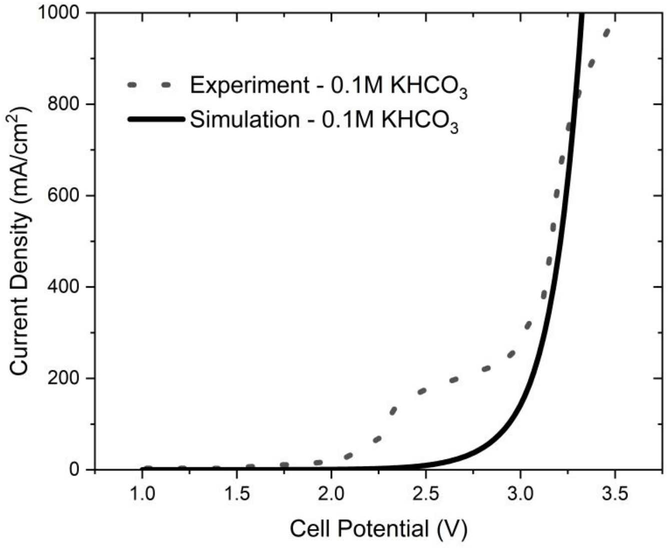

Figure S4: experimental polarisation curve vs simulated polarisation curve of base case simulation presented in this work, using the i_0/α pairs in Table 2.

Input Parameters as Sources of Infidelity in Multiphysics Simulations:

In the simulations summarised by Table 1, the values prescribed to the solution phase equilibrium constants and reaction rate constants (K_{eq}, k_f) are those found in Schulz [2] et al., which can be traced back to the work of Eigen [3] et al. Sometimes these values are rounded to the nearest order of magnitude. Schulz et al. originally determine these rate constants in sea water system, conditions which different from that of an electrochemical cell during operation. Given that the rate constant and equilibrium constants can change in local environments and under electric fields[1], they are a possible source of infidelity to simulations of this type. Alternatively, one may calculate K_{eq} as in Weng[4] et al., using thermodynamic information.

Values for liquid phase diffusion coefficients (D_i) used are generally those found in Lide [5] et al. As the assumption of infinite dilution and no ion-ion interactions may be unsuitable for CO_2 electrochemical reactors, these parameters are also a possible source of infidelity.

Values for the active surface area of porous electrodes ($a_{cathode/anode}$), conductivities of the electrodes ($\sigma_{anode/cathode}$), fixed charge of the membrane (ρ_{mem}), and the porosities ($\square_{anode/cathode/membrane}$), can be calculated using information in literature which studies porous electrode/membrane properties[6,7]. However, the exact numbers will differ between experimental setups if not using the exact same materials. Thus these parameters are possible source of infidelity too. Further, the hydrogen evolution reaction can occur not just on the catalysts layer, which is deposited upon carbon substrate, but also on the carbon substrate itself[8], introducing further infidelity as this is not discussed or accounted for in published simulations.

Sensitivity Analysis Data:

Table S1: Sensitivity data for 1% perturbation at 10mA/cm^2 (data composing Figure 6):

**Parameter**	**% change in CO current density, for the $KHCO_3$ case**	**% change in CO current density, for the KOH case**
$\alpha_{c,1}$	2.43	2.68
$\alpha_{c,2}$	-1.02	-1.04
$\alpha_{a,3}$	5.29	6.01
$i_{0,1}$	0.47	0.52
$i_{0,2}$	-0.08	-0.08
$i_{0,3}$	0.28	0.32
k_4	0.13	0.01
k_5	-1.83E-04	0.12
k_6	0.12	0.14
k_7	-1.65E-06	-1.29E-08
k_8	1.03E-05	0.03
$K_{eq,4}$	-0.12	1.28E-06
$K_{eq,5}$	-9.93E-04	-0.12
$K_{eq,6}$	5.31E-05	2.54E-05
$K_{eq,7}$	0.12	0.03
$K_{eq,8}$	7.85E-13	-4.45E-11
Area of cell	4.97E-13	1.38E-11
Pore radius	1.14E-06	1.61E-06
Thin film thickness	-5.34E-06	-2.88E-04
Length (cathode)	0.49	0.56

Length (membrane)	-0.03	-0.03
Length (anode)	0.39	0.57
Conductivity (cathode)	4.50E-04	4.59E-04
Conductivity (anode)	6.65E-05	9.42E-05
Porosity (cathode)	0	0
Porosity (membrane)	0.05	0.08
Porosity (anode)	-1.54E-04	-2.18E-04
Specific surface area (cathode)	0.39	0.44
Specific surface area (anode)	0.28	0.32
Fixed charge density (membrane)	0.02	0.02
x_{CO2}	0.14	0.18
D_{CO2}	7.67E-05	0.04
D_{H+}	0.01	1.84E-06
D_{OH-}	0	-0.06
D_{HCO3-}	0.04	-0.01
D_{CO3--}	0.01	-0.03
D_{K+}	6.41E-11	-1.21E-08
D_{H2O}	1.33E-05	-4.59E-05
D_{CO-CO2}	2.79E-07	1.01E-07
D_{CO-N2}	2.95E-06	3.79E-06
D_{H2-CO}	9.13E-13	-2.52E-11
D_{H2-CO2}	3.93E-10	1.41E-11
D_{H2-N2}	4.20E-12	-3.68E-11
D_{N2-CO2}	4.86E-07	1.37E-06

Table S2: Sensitivity data for 1% perturbation at 1000mA/cm² (data composing Figure 6):

Parameter	% change in CO current density, for the KHCO3 case	% change in CO current density, for the KOH case
$\alpha_{c,1}$	2.43	2.68
$\alpha_{c,2}$	-1.02	-1.04
$\alpha_{a,3}$	5.29	6.01
$i_{0,1}$	0.47	0.52
$i_{0,2}$	-0.08	-0.08
$i_{0,3}$	0.28	0.32
k_4	0.13	0.01
k_5	-1.83E-04	0.12
k_6	0.12	0.14
k_7	-1.65E-06	-1.29E-08
k_8	1.03E-05	0.03
$K_{eq,4}$	-0.12	1.28E-06
$K_{eq,5}$	-9.93E-04	-0.12
$K_{eq,6}$	5.31E-05	2.54E-05
$K_{eq,7}$	0.01	0.03
$K_{eq,8}$	7.85E-13	-4.45E-11
Area of cell	4.97E-13	1.38E-11
Pore radius	1.14E-06	1.61E-06
Thin film thickness	-5.34E-06	-2.88E-04
Length (cathode)	0.49	0.56

Length (membrane)	-0.03	-0.03
Length (anode)	0.39	0.57
Conductivity (cathode)	4.50E-04	4.59E-04
Conductivity (anode)	6.65E-05	9.42E-05
Porosity (cathode)	0	0
Porosity (membrane)	0.05	0.08
Porosity (anode)	-1.54E-04	-2.18E-04
Specific surface area (cathode)	0.39	0.44
Specific surface area (anode)	0.28	0.32
Fixed charge density (membrane)	0.02	0.02
x_{CO2}	0.14	0.18
D_{CO2}	7.67E-05	0.04
D_{H+}	0.01	1.84E-06
D_{OH-}	0	-0.06
D_{HCO3-}	0.04	-0.01
D_{CO3--}	0.01	-0.03
D_{K+}	6.41E-11	-1.21E-08
D_{H2O}	1.33E-05	-4.59E-05
D_{CO-CO2}	2.79E-07	1.01E-07
D_{CO-N2}	2.95E-06	3.79E-06
D_{H2-CO}	9.13E-13	-2.52E-11
D_{H2-CO2}	3.93E-10	1.41E-11
D_{H2-N2}	4.20E-12	-3.68E-11
D_{N2-CO2}	4.86E-07	1.37E-06

Sensitivity Analysis Performed with 0.01% Perturbation:

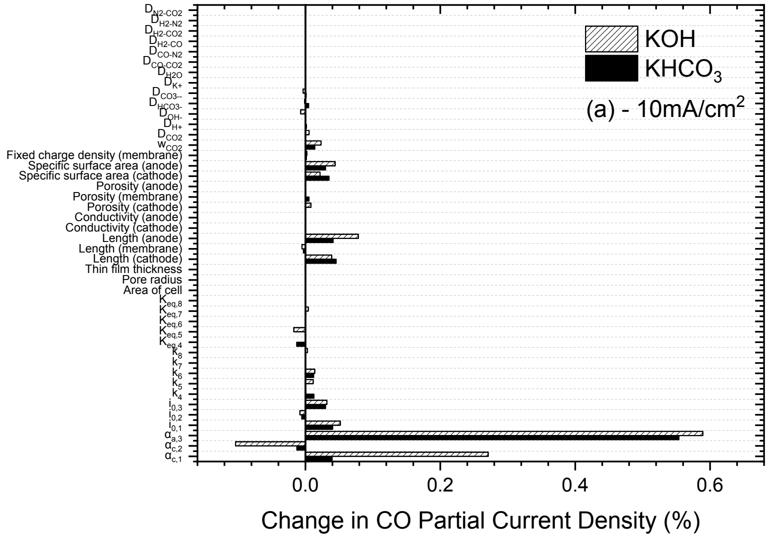

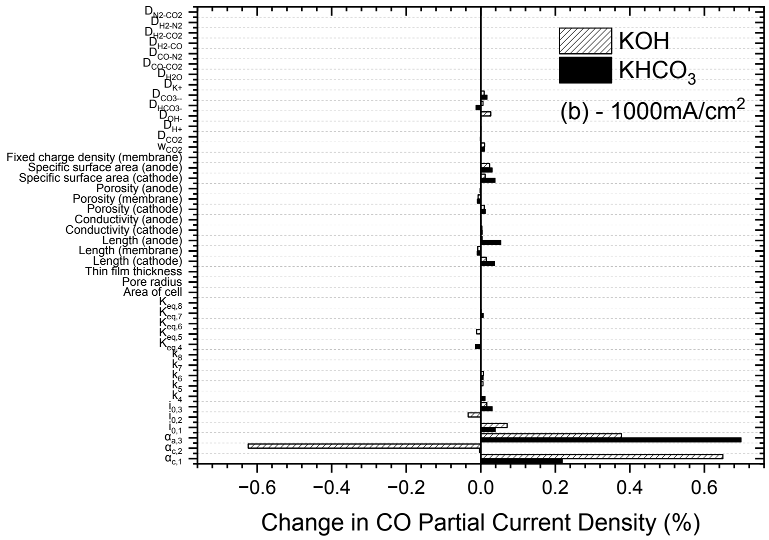

Figure S5: Sensitivity analysis for 0.01% perturbation of each parameter, at (a) 10mA/cm² and (b) 1000mA/cm².

Reactions Not Considered in Multiphysics Simulations for CO_2 Reduction:

$$H_2CO_3 \rightleftharpoons H^+ + HCO_3^-$$

$$H_2CO_3 \rightleftharpoons H_2O + CO_2$$

$$K_2CO_3 \rightleftharpoons K+ + KCO_3-$$

$$KCO_3^- \rightleftharpoons K^+ CO_3^{2-}$$

$$KOH \rightleftharpoons K^+ + OH^-$$

$$KHCO_3 \rightleftharpoons K^+ + HCO_3^-$$

$$KHCO_3 \rightleftharpoons H^+ + KCO_3^-$$

The above reactions are not considered in multiphysics simulations but are likely occurring in the cell if KOH or $KHCO_3$ anolyte is used. Future computational works may wish to determine

whether the occurrence of these reactions is significantly impacting cell performance. Such work would necessitate the gathering of kinetic data for these reactions.

Values of Aqueous Species in Anolyte Solution:

**Species:**	**Concentration (0.1M $KHCO_3$ case)**	**Concentration (0.1M KOH case)**
H_2O	55M	55M
K^+	0.1M	0.1M
OH^-	1e-6M	0.1M
HCO_3^-	0.1M	1e-9M
H^+	1e-8M	1e-13M
CO_3^{2-}	1e-6M	1e-7M

Discussion On Error Propagation Due to Network of Reactions:

The concentrations of any species in the system are tightly linked to the concentration of all other species, through the reaction network of solution phase and electrochemical reactions. Thus, any infidelity in the simulation to one concentration will propagate to the other concentrations, and affect other simulation results e.g., current densities, Faradaic efficiencies. It is impossible to isolate the effects of one reaction for this reason - even if we change only the rate constant of one reaction, the rates of all other reactions will be affected.

Figure S5 below also informs us of which species may play the significant roles in device performance – the OH^- ion for instance, which has the largest number of connections. OH^- affects cell performance primarily through Reaction 6, whose rate we should aim to minimize [9].

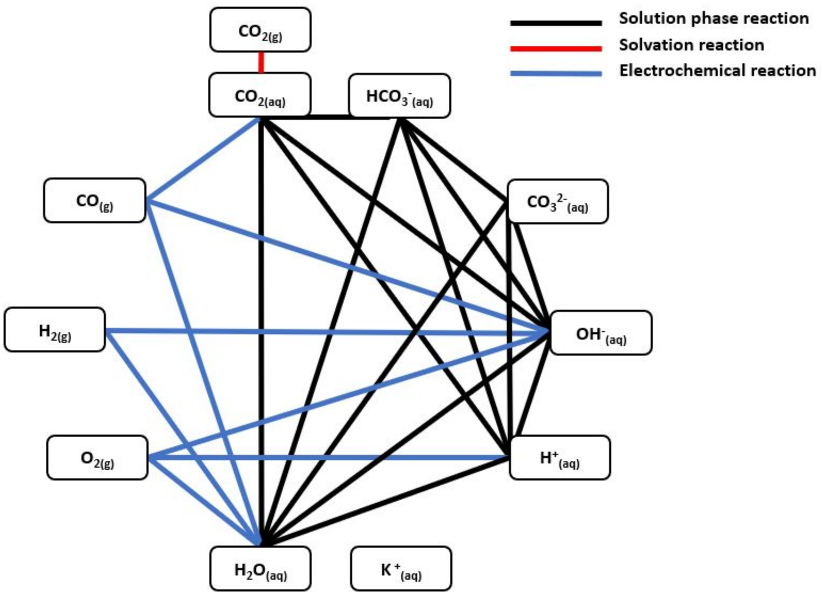

Figure S6: Reaction network in the simulation presented in this work. Each species (node) is connected to each other species through chemical reactions (solution phase, electrochemical, or solvation). K^+ is treated as a free species which is not involved in any reactions.

References:

1. Bui, J. C.; Lees, E. W.; Pant, L. M.; Zenyuk, I. V.; Bell, A. T.; Weber, A. Z., Continuum Modeling of Porous Electrodes for Electrochemical Synthesis. *Chem Rev* **2022,** *122* (12), 11022-11084.
2. Schulz, K. G.; Riebesell, U.; Rost, B.; Thoms, S.; Zeebe, R. E., Determination of the rate constants for the carbon dioxide to bicarbonate inter-conversion in pH-buffered seawater systems. *Marine Chemistry* **2006,** *100* (1-2), 53-65.
3. EIGEN, M., Proton Transfer, Acid-Base Catalysis, and Enzymatic Hydrolysis. *ANGEWANDTE CHEMIE* **1964,** *3*, 1-72.
4. Weng, L.-C.; Bell, A. T.; Weber, A. Z., Towards membrane-electrode assembly systems for CO2 reduction: a modeling study. *Energy & Environmental Science* **2019,** *12* (6), 1950-1968.
5. Lide, D. R., *CRC Handbook of Chemistry and Physics*. CRC Press: Boca Raton, FL, 2005.
6. Soboleva, T.; Zhao, X.; Malek, K.; Xie, Z.; Navessin, T.; Holdcroft, S., On the micro-, meso-, and macroporous structures of polymer electrolyte membrane fuel cell catalyst layers. *ACS Appl Mater Interfaces* **2010,** *2* (2), 375-84.
7. Peng, J.; Roy, A. L.; Greenbaum, S. G.; Zawodzinski, T. A., Effect of CO2 absorption on ion and water mobility in an anion exchange membrane. *Journal of Power Sources* **2018,** *380*, 64-75.
8. Yang, K.; Kas, R.; Smith, W. A.; Burdyny, T., Role of the Carbon-Based Gas Diffusion Layer on Flooding in a Gas Diffusion Electrode Cell for Electrochemical CO2 Reduction. *ACS Energy Letters* **2020,** *6* (1), 33-40.
9. Rabinowitz, J. A.; Kanan, M. W., The future of low-temperature carbon dioxide electrolysis depends on solving one basic problem. *Nat Commun* **2020,** *11* (1), 5231.
